# Prognostic Value of Long Non-Coding RNA *HOTAIR* in Various Cancers

**DOI:** 10.1371/journal.pone.0110059

**Published:** 2014-10-10

**Authors:** Qiwen Deng, Huiling Sun, Bangshun He, Yuqin Pan, Tianyi Gao, Jie Chen, Houqun Ying, Xian Liu, Feng Wang, Yong Xu, Shukui Wang

**Affiliations:** 1 Central Laboratory, Nanjing First Hospital, Nanjing Medical University, Nanjing, Jiangsu, China; 2 Department of Life Sciences, Nanjing Normal University, Nanjing, Jiangsu, China; 3 Department of Clinical Laboratory, Nanjing First Hospital, Nanjing Medical University, Nanjing, Jiangsu, China; 4 Medical college, Southeast University, Nanjing, Jiangsu, China; Sanjay Gandhi Medical Institute, India

## Abstract

Long non-coding RNA has been involved in cancer progression, and high HOX transcript antisense intergenic RNA (*HOTAIR*) is thought to be a poor prognostic indicator in tumorigenesis of multiple types of cancer. Hence, the present study further reveals its prognostic value in tumor malignancy. A systematic review of PubMed and Web of Science was carried out to select literatures relevant to the correlation between *HOTAIR* expression levels and clinical outcome of various tumors. Overall survival (OS), metastasis-free survival (MFS), recurrence-free survival (RFS), and disease-free survival (DFS) were subsequently analyzed. Data from studies directly reporting a hazard ratio (HR) and the corresponding 95% confidence interval (CI) or a *P* value as well as survival curves were pooled in the current meta-analysis. A total of 2255 patients from 19 literatures almost published in 2011 or later were included in the analysis. The results suggest that *HOTAIR* was highly associated with HR for OS of 2.33 (95%CI = 1.77-3.09, *P*_heterogeneity_ = 0.016). Stratified analyses indicate that elevated levels of *HOTAIR* appears to be a powerful prognostic biomarker for patients with colorectal cancer (HR = 3.02, 95CI% = 1.84-4.95, *P*_heterogeneity_ = 0.699) and esophageal squamous cell carcinomas (HR = 2.24, 95CI% = 1.67-3.01, *P*_heterogeneity_ = 0.711), a similar effect was also observed in analysis method and specimen, except for ethnicity. In addition, Hazard ratios for up-regulation of *HOTAIR* for MFS, RFS, and DFS were 2.32 (*P*<0.001), 1.98 (*P* = 0.369), and 3.29 (*P* = 0.001), respectively. In summary, the high level of *HOTAIR* is intimately associated with an adverse OS in numerous cancers, suggesting that *HOTAIR* may act as a potential biomarker for the development of malignancies.

## Introduction

Noncoding RNAs (ncRNA) are initially identified from sequencing and microarray for whole genome and transcriptome, and at least 90% of ncRNAs has been found to be actively transcribed [1], [2]. The transcription of ncRNA revealed its complication in biogenesis than protein-coding RNA, such as extensive antisense, overlapping and non-coding RNA expression [3], [4], [5]. Despite initial argument claimed that ncRNA may be a fake transcriptional noise, increasing evidences suggested that ncRNAs may play a dominant biological role in cell metabolism and survival [6], [7], [8], [9]. Furthermore, the recent studies demonstrated that long non-coding RNAs (lncRNA, 200nt in length) express at tissue-specific patterns and that it is abnormally regulated in a variety of diseases, including cancer [10], [11], [12], [13]. Multiple regulatory bases have been involved in the regulation of lncRNA, such as transcriptional regulation, epigenetic regulation, and posttranscriptional regulation [9], [14]. Moreover, lncRNAs exhibit unique profiles in many kinds of cancers, which represent carcinogenesis and progression regarded as a predictor of patient outcomes [15], [16], [17].

*HOTAIR*, a prominently focused lncRNA, was initially reported to be implicated in primary breast cancer and breast cancer metastasis, wherein elevated *HOTAIR* promoted tumor invasiveness and metastasis [18]. *HOTAIR* overexpression has been shown to be associated with expression of polycomb repressive complex 2 (PRC2), inducing its relating methylation of histone H3 lysine 27 (H3K27) [10], [18]. In addition to breast cancer [18], recent clinical evidences show *HOTAIR* is also involved in the progression of many other types of cancer, such as hepatocellular carcinomas (HCC) [19], colorectal cancer (CRC) [20], esophageal squamous cell carcinomas (ESCC) [21], suggesting that *HOTAIR* expression serves as a prognostic factor for tumorigenesis. Although *HOTAIR* expression is considered to relating to clinical prognosis of multiple cancers, the impact of *HOTAIR* on the development of cancer still remains elusive. Some studies reported that up-regulation of *HOTAIR* contributes to tumorigenesis, including bladder cancer [22], cervical cancer [23], colorectal cancer [24], etc., while a few evidences exhibited an adverse effect recognized as a protective factor to against carcinogenesis [25]. It is necessary therefore to clarify the relationship between *HOTAIR* and cancer. Thus, the present study conducted the first meta-analysis using qualified relevant literatures to achieve a precise evaluation of the association between *HOTAIR* expression and cancer clinical prognosis.

## Materials and Methods

### Data sources and searches

The published data searching was performed using a literature review system with the Preferred Reporting Items for Systematic Reviews and Meta-Analysis guidelines [26]. The selected literatures were determined via an electronic search of PubMed and Web of Science using these following terms: “*HOTAIR*”, “cancer or tumor or carcinomas” and “prognosis or outcome”. The last search was updated in July 18, 2014. Citation lists of retrieved articles were searched manually to ensure sensitivity of the search strategy.

### Study selection

Studies considered eligible met the following criteria: 1) studied patients with any type of cancers; 2) explored the link between *HOTAIR* and clinical prognosis; 3) availability of a hazard ratio (HR) and 95% confidence interval (CI) or a *P* value for overall survival (OS). For a secondary analysis, studies including an HR for metastasis-free survival (MFS), disease-free survival (DFS), or recurrence-free survival (RFS) were also used to further analyze. OS [27], MFS [28], DFS [27], and RFS [29] were described previously; 4) published as a full paper in English. Studies were excluded based on the following criteria: 1) duplicated studies, reviews, letters, unpublished data, and comments; 2) those published in language other than English; 3) lack of key information for further analysis; 4) non-human research.

### Data extraction

Two investigators (QWD, HLS) independently evaluated and extracted data from each identified studies based on criteria of inclusion and exclusion. Corresponding authors were contacted to clarify missing or ambiguous data. OS was treated as a dominant outcome of interest, but MFS, RFS and DFS were set as the secondary outcomes. The following information was carefully extracted: name of first author, year of publication, country of origin, ethnicity of the study population, type of specimen, cancer type, number of patients included in analysis, detection method of *HOTAIR*, cut-off defining high *HOTAIR*, follow-up period, and HR and corresponding 95% CI for OS, MFS, RFS, or DFS as applicable. Cancer type subgroups were generated for the main outcome if at least two studies on the type of cancer were available; the only one study was pooled in a subgroup termed “Other.” HR was firstly extracted from multivariable analysis where available. Otherwise, HR was extracted from univariate analysis, and calculated from Kaplan-Meier survival curve by HR digitizer software Engauge 4.0 as described previously [30].

### Statistical analyses

All extracted data were combined into a meta-analysis using STATA software version 11.0 (STATA Corporation, College Station, TX, USA). Hazard ratios with the corresponding 95% CIs were used to estimate the strength of the link between *HOTAIR* and clinical prognosis. If HR was not directly reported, a mathematical estimation was conducted by calculating the necessary data on the basis of the previously reported methods [31]. Cochran's Q test and Higgins I-squared statistic were used to estimate the heterogeneity of pooled results. If *P* <0.05 for Q-test showed significant heterogeneity among studies, the random-effects model (DerSimoian-Laird method) was implemented to calculate the pooled HRs [32]. Otherwise, the fixed-effects model (Mantel-Haenszel method) was used [33]. To further explore the potential source of heterogeneity among studies, meta-regression was performed utilizing variables as cancer type, ethnicity, analysis method, type of specimen. To validate the stability of outcomes in this meta-analysis, sensitivity analysis was performed by sequential omission of each individual study. Publication bias was conducted by Begg's funnel plot and Egger's linear regression test and a *P* <0.05 was considered representative of statistically significant publication bias.

## Results

### Characteristics of studies

**Figure 1:**
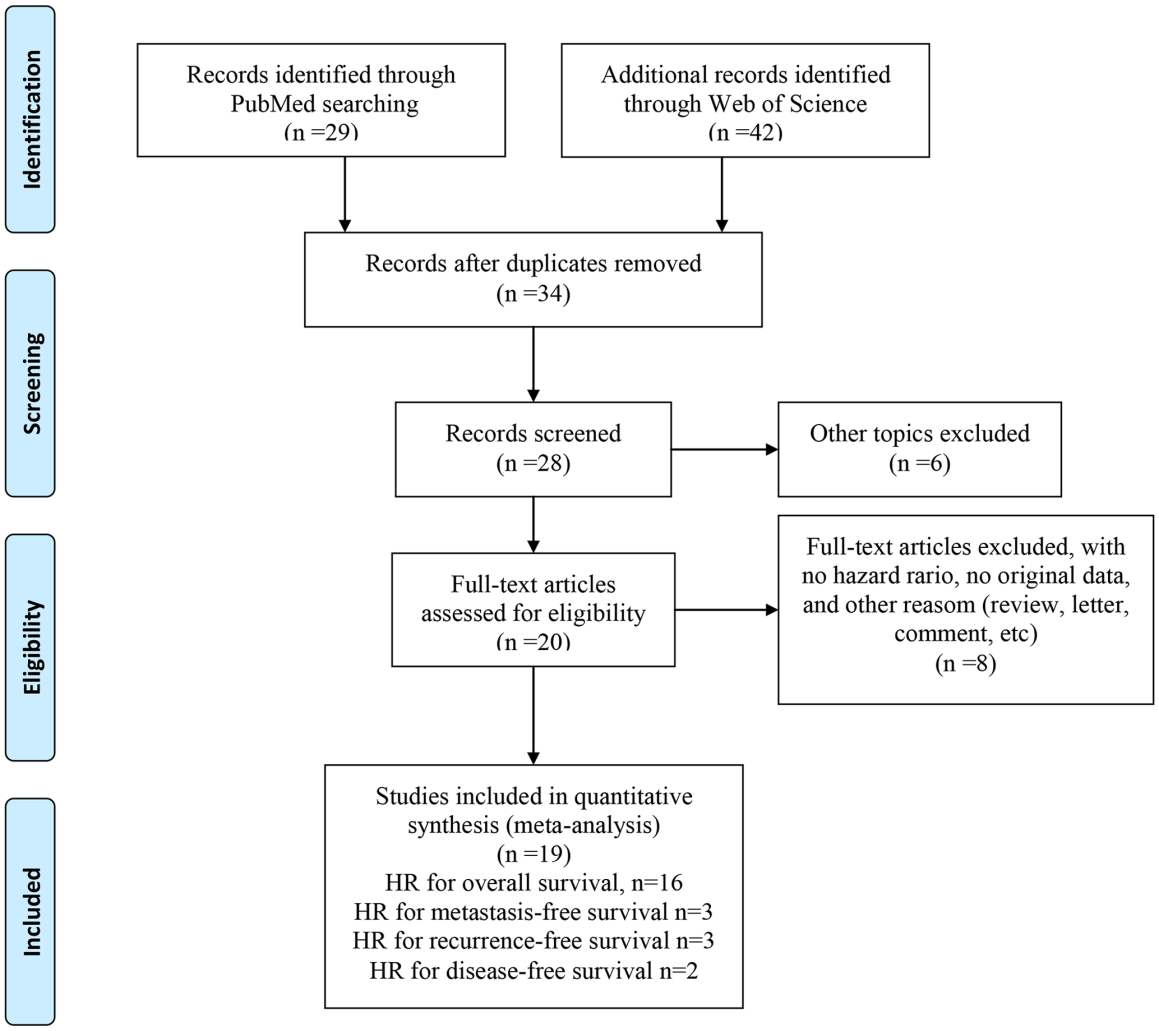
Flow chart for selection of studies for inclusion in this meta-analysis.

There were 71 papers in the electronic search of PubMed and EMBASE. On the basis of the inclusion criteria, 19 eligible papers were enrolled in this meta-analysis (shown in Figure 1). The main characteristics of included studies are shown in Table 1; all studies were almost published in 2011 or later. There were 16 studies for OS, 3 for MFS, 3 for RFS, and 2 for DFS in the meta-analysis. Participants in 16 studies were Asian and in the other 3 studies were Caucasian. Various cancers were recorded in our study, including BC, HCC, CRC, ESCC, etc. The types of specimen were tissue for twenty-two studies and blood for one study. The cut-off values included in the studies were inconsistent due to different detection methods, even in 6 studies were not reported. Hazard ratios with the corresponding 95% CIs were extracted from univariate analysis and the graphical survival plots in 6 studies, and multivariate analysis in 18 studies.

**Table 1:** Main characteristics of included studies.

First author	Year	Country	Ethnicity	Specimens	Cancer type	Method	Cut-off*	Follow-up (month)	Number	Analysis	Survival
Yang [19]	2011	China	Asian	Tissue	HCC	RT-qPCR	NR	18.6 (median)	60	Multivariable	RFS
Kogo [20]	2011	Japan	Asian	Tissue	CRC	RT-qPCR	0.027	36 (mean)	100	Multivariable	OS
Lu [40]	2012	Italy	Caucasian	Tissue	BC	RT-qPCR	NR	86 (median)	336	Multivariable	OS, RFS
Li [41]	2012	China	Asian	Tissue	LSCC	RT-qPCR	NR	60 (total)	72	Multivariable	OS
Nie [42]	2013	China	Asian	Tissue	NPC	ISH	6	69 (median)	160	Multivariable	OS
Nakagawa [43]	2013	Japan	Asian	Tissue	NSCLC	RT-qPCR	2	31.4 (median)	77	Univariable	DFS
Lv [44]	2013	China	Asian	Tissue	ESCC	ISH	6	60 (total)	93	Univariable	OS
Li [45]	2013	China	Asian	Tissue	ESCC	RT-qPCR	125	60 (total)	100	Multivariable	OS
Ge [46]	2013	China	Asian	Tissue	ESCC	RT-qPCR	NR	60 (total)	137	Multivariable	OS, MFS
Liu [47]	2013	China	Asian	Tissue	NSCLC	RT-qPCR	8.57	60 (total)	42	Univariable	OS
Chen [48]	2013	China	Asian	Tissue	ESCC	RT-qPCR	26.6	38 (mean)	78	Multivariable	OS
Zhang [49]	2013	China	Asian	Tissue	GBM	GSEA	NR	50 (totoal)	89	Multivariable	OS
Sørensen [50]	2013	Denmark	Caucasian	Tissue	BC	Microarray	0.6	217.2 (mean)	164	Multivariable	MFS
Svoboda [51]	2014	Czech	Caucasian	Tissue, Blood	CRC	RT-qPCR	0.7, 4.4	35 (mean)	73, 84	Multivariable	OS
Qiu [52]	2014	China	Asian	Tissue	EOC	RT-qPCR	NR	50.5 (median)	64	Univariable	OS, DFS
Huang [53]	2014	China	Asian	Tissue	Cervical	RT-qPCR	NR	42 (mean)	218	Multivariable	OS
Liu [54]	2014	China	Asian	Tissue	GC	RT-qPCR	NR	50 (totoal)	78	Univariable	OS
Wu [55]	2014	China	Asian	Tissue	CRC	RT-qPCR	5	55.5 (median)	120	Multivariable	OS, MFS
Yan [29]	2014	China	Asian	Tissue	Bladder	RT-qPCR	NR	60 (total)	110	Multivariable	RFS

*cancerous/noncancerous

HCC, hepatocellular carcinoma; CRC, colorectal cancer; BC, breast cancer; LSCC, laryngeal squamous cell carcinoma; NPC, nasopharyngeal carcinoma; NSCLC, non-small cell lung cancer; ESCC, esophageal squamous cell carcinoma; GBM, glioblastoma multiforme; EOC, epithelial ovarian cancer; GC, gastric cancer; RT-qPCR, real-time quantitative PCR; ISH, in situ hybridization; GSEA, gene set enrichment analysis; NR, not reported; OS, overall survival; MFS, metastasis-free survival; RFS, recurrence-free survival; DFS, disease-free survival.

### Overall survival

**Figure 2:**
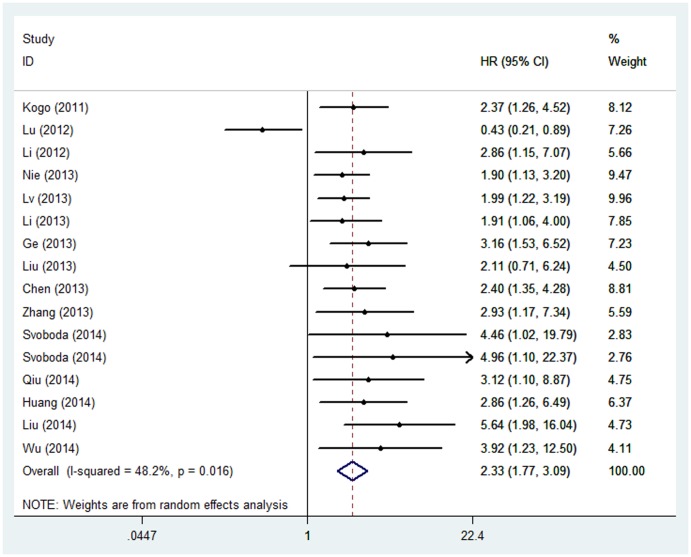
Forest plots of studies evaluating hazard ratios (HRs) of *HOTAIR* for overall survival.

The main results of this meta-analysis are shown in Table 2. 16 studies comprising 1844 patients reported HR for OS. It is suggested that elevated *HOTAIR* predicted a poor outcome for OS (HR = 2.33, 95%CI  = 1.77-3.09, *P*_H_ = 0.016; Figure 2). Stratified analyses by cancer type indicated that the prognostic effect of *HOTAIR* was highest in CRC (HR = 3.02, 95%CI = 1.84-4.95, *P*_H_ = 0.699), followed by ESCC (HR = 2.24, 95%CI = 1.67-3.01, *P*_H_ = 0.016). HR for the subgroup of other cancers was 2.18 (95%CI = 1.25-3.78, *P*_H_ =  0.001).

**Table 2:** The main results of pooled analyses.

Survival	Variables	NO. of studies	NO. of patients	*P* value			Regression model	
				*P*_H_	*P*_Z_	*P*_E_	Random	Fixed
OS	All	16	1844	0.016	0.000	0.110	***2.34 (1.77–3.09)***	2.20 (1.82–2.66)
	Cancer type							
	CRC	4	377	0.699	0.000	—	3.02 (1.84–4.95)	***3.02 (1.84–4.95)***
	ESCC	4	408	0.711	0.000	—	2.24 (1.67–3.01)	***2.24 (1.67–3.01)***
	Other	8	1059	0.001	0.006	—	***2.18 (1.25–3.78)***	1.95 (1.46–2.60)
	Ethnicity							
	Asian	13	1351	0.894	0.000	—	2.43 (1.99–2.97)	***2.43 (1.99–2.97)***
	Caucasian	3	493	0.001	0.482	—	*1.92 (0.31–11.91)*	0.92 (0.51–1.67)
	Analysis method							
	Univariable	4	277	0.329	0.000	—	2.53 (1.64–3.89)	***2.13 (1.71–2.65)***
	Multivarible	12	1567	0.009	0.000	—	***2.26 (1.59–3.20)***	2.44 (1.67–3.55)
	Specimen							
	Tissue	15	1760	0.015	0.000	—	***2.29 (1.72–3.04)***	2.17 (1.79–2.63)
	Blood	1	84	—	0.037	—	4.96 (1.10–22.37)	***4.96 (1.10–22.37)***
MFS	All	3	421	0.080	0.000	—	2.81 (1.44–5.57)	***2.32 (1.62–3.33)***
RFS	All	3	506	0.000	0.369	—	*1.98 (0.44–8.85)*	1.96 (1.37–2.81)
DFS	All	2	141	0.969	0.001	—	3.29 (1.61–6.70)	***3.29 (1.61–6.70)***

*P*_H_, *P* value of heterogeneity test; *P*_Z_, *P* value of Z test; *P*_E_, *P* value of Egger's test; CRC, colorectal cancer; ESCC, esophageal squamous cell carcinoma; OS, overall survival; MFS, metastasis-free survival; RFS, recurrence-free survival; DFS, disease-free survival.

Italic indicates HR with 95% CI used to analyses.

The bold represents statistically significant results.

The effect of elevated *HOTAIR* on OS among different races is shown in Table 2. The hazard ratios were 2.43 (95%CI = 1.99-2.97, *P*_H_ =  0.894) for Asian, and 1.92 (95%CI = 0.31-11.91, *P*_H_ =  0.001) for Caucasian. When different analysis methods were considered, *HOTAIR* was a strong prognostic marker both by univariate analysis (HR =  2.13, 95%CI = 1.71-2.65, *P*_H_ = 0.329) and by multivariate analysis (HR = 2.26, 95%CI = 1.59-3.20, *P*_H_ = 0.009). Performing subgroup analyses stratified by specimen, increased *HOTAIR* was closely associated with poor prognosis both in tissue (HR = 2.29, 95%CI = 1.72-3.04, *P*_H_ = 0.015) and in blood (HR = 4.96, 95%CI = 1.10-22.37).

**Figure 3:**
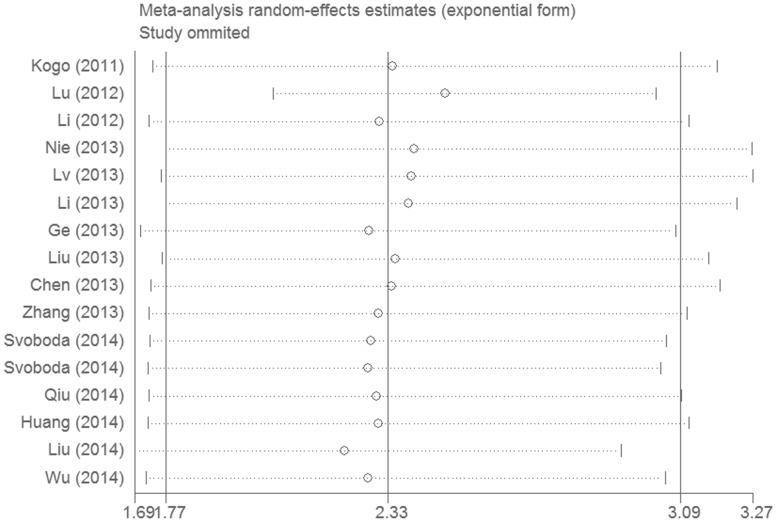
Sensitivity analysis of effect of individual studies on the pooled HRs for *HOTAIR* and overall survival of patients.

**Figure 4:**
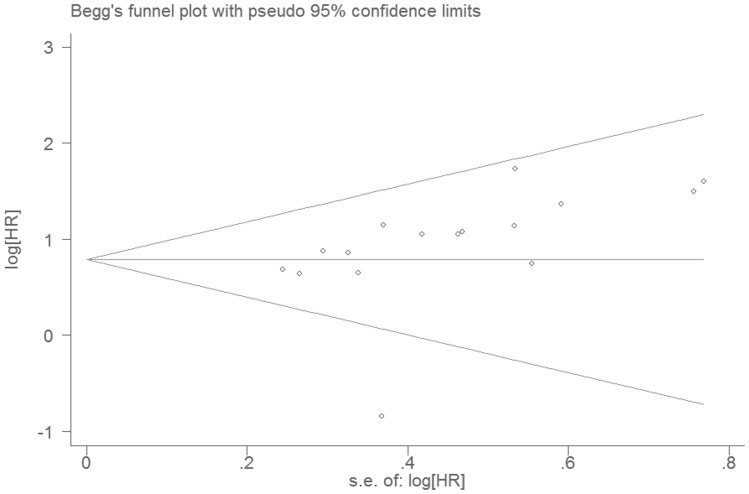
Funnel plot of HR for overall survival for high *HOTAIR* (vertical axis) and the standard error (SE) for HR (horizontal axis). Each study represented by one circle. The horizontal line represented the pooled effect estimate.

Sensitivity analysis is presented in Figure 3. The result pattern was not significantly impacted by removing single study each time. Begg's funnel plot and the Egger's linear regression test were conducted to evaluate publication bias. The shape of the funnel plot showed no significant asymmetry in Figure 4. Subsequently, Egger's test also suggested no evidence of publication bias (*P* = 0.110).

### Metastasis-free survival, recurrence-free survival and disease-free survival

Three studies comprising 421 patients reported HRs for MFS. Overall, *HOTAIR* greater than the cut-off was associated with an HR for MFS of 2.32 (95%CI = 1.62-3.33, *P*_H_ = 0.080). Three studies comprising 506 patients showed HRs for RFS. *HOTAIR* was not linked with poor RFS. Two studies comprising 141 patients reported HRs for DFS. Up-regulation of *HOTAIR* predicted a poor clinical outcome for DFS (HR = 3.29, 95%CI = 1.61-6.70, *P*_H_ = 0.969).

## Discussion

As a novel molecular basis, the study of lncRNA has focused on the impact of lncRNA on cancer pathogenesis and prognosis, providing a new insight into cancer therapeutic strategy [34], [35]. Despite substantial progress of lncRNAs in cancer nosogenesis and prognosis, the prognostic effect of lncRNAs is still confused. To explore the prognostic impact of lncRNAs in cancer, this system review and meta-analysis was performed to investigate the impact of *HOTAIR* on tumor prognosis for achieving more consistent and precise conclusion.

Here we undertook meta-analysis of 18 literatures comprising 2255 patients with tumors to assess the prognostic effect of *HOTAIR*. We found that an accordant effect of an elevated *HOTAIR* on OS (HR = 2.33) with the similar hazard ratios among various cancer type subgroups and across analytical methods, or specimens, except for ethnicity subgroups. *HOTAIR* has been shown to contribute to the progression of many types of cancer and is nowadays considered as a hallmark of cancer [36]. The strong impact on OS was highest in CRC, which is further supported by evidences that *HOTAIR* plays a critical role in the carcinogenesis of CRC as a result of promoted multipotent cell differentiation [20]. In particular, the elevated levels of *HOTAIR* are highly associated with worse OS in Asian, but not in Caucasian, suggesting that interaction between genetic and environmental factors may contribute to cancer development. In addition, clinicopathological features and drug treatment status could modify the effect of *HOTAIR* [25]. However, a study reported that *HOTAIR* induces repressive chromatin status by promoting the formation of H3K27, suggesting that *HOTAIR* may function as a tumor suppressor gene by inhibiting proliferation of cancer stem cells [18]. The present study further verify that the levels of *HOTAIR* is highly associated with cancer development, although the prognostic effect of *HOTAIR* on MFS and DFS is still remained for the future studies. Importantly, the results are consistent in the tested types of cancer regardless of different data resources and analysis methods.

The mechanisms underlying the association between the high levels of *HOTAIR* and poor outcome of cancers are still unclear. This study may predict the following potential mechanisms involved in the prognostic impact of *HOTAIR* on carcinogenesis. *HOTAIR* regulates the chromatin methylation state by inducing genome-wide retargeting of PRC2 and promotes metastasis of breast cancer by silencing multiple metastasis-suppressing genes [18]. Consequently, PRC2 has been reported to be involved in stem cell pluripotency and progression ofEZH2, SUZ12, and EED [36], [37]. Consistently, *HOTAIR* knockdown not only suppressed cell invasion, but also decreased cell proliferation, altered cell cycle progression, and induced apoptosis [38].

Physicians prefer to use prognostic data when speaking to patients. As *HOTAIR* offers independent prognostic information, we may incorporate *HOTAIR* in a simple score to provide an appropriate therapeutic strategy. In recent years, a few clinical studies with cancer patients showed that reducing levels of *HOTAIR* is closely associated with a good response to treatment [39]. Furthermore, *HOTAIR* has been verified to be an independent prognostic indicator in CRC patients [24]. It was suggested that changing blood *HOTAIR* levels might be useful for tailoring of therapy for cancer patients.

Some limitations in this study should be acknowledged. Firstly, only summarized data rather than individual patient data were used. Secondly, a part of studies, especially in subgroups analyses, was lightly relative. Thirdly, because some cut-off values were not reported and the criteria of calculating cut-off value were inconsistent, stratified analysis by cut-off values was not conducted to suggest whether cut-off values were the origin of heterogeneity. Fourthly, we only included studies reporting HR or survival curves, and consequently some publications reporting on the prognostic value of *HOTAIR* were excluded. For example, only odds ratios were reported, so the selection bias might be appeared. Finally, most of the included studies do not explicitly control for such concurrent conditions, and these may confound the measurement of *HOTAIR*.

In summary, up-regulation of *HOTAIR* is associated with adverse survival in many types of cancer, and *HOTAIR* may serve as an effective prognostic biomarker for diagnosis of cancer. Therefore, clinical checking the levels of *HOTAIR* expression may provide a promising approach to identify patients who would require more intimately care for personally tailored medical inspection to monitor cancer prevention and treatment.

## Supporting Information

Figure S1 **Flow chart.**
(TIF)

Figure S2 **Forest plots.**
(TIF)

Figure S3 **Sensitivity analysis.**
(TIF)

Figure S4 **Funnel plot.**
(TIF)

Table S1 **Main characteristics.**
(XLSX)

## References

[1] Birney E, Stamatoyannopoulos JA, Dutta A, Guigo R, Gingeras TR, et al (2007) Identification and analysis of functional elements in 1% of the human genome by the ENCODE pilot project. Nature 447: 799–816. 17571346 10.1038/nature05874 PMC2212820

[2] Costa FF (2010) Non-coding RNAs: Meet thy masters. Bioessays 32: 599–608. 20544733 10.1002/bies.200900112

[3] Kapranov P, Willingham AT, Gingeras TR (2007) Genome-wide transcription and the implications for genomic organization. Nat Rev Genet 8: 413–423. 17486121 10.1038/nrg2083

[4] Frith MC, Pheasant M, Mattick JS (2005) The amazing complexity of the human transcriptome. Eur J Hum Genet 13: 894–897. 15970949 10.1038/sj.ejhg.5201459

[5] Guttman M, Amit I, Garber M, French C, Lin MF, et al (2009) Chromatin signature reveals over a thousand highly conserved large non-coding RNAs in mammals. Nature 458: 223–227. 19182780 10.1038/nature07672 PMC2754849

[6] Wang J, Zhang J, Zheng H, Li J, Liu D, et al. (2004) Mouse transcriptome: neutral evolution of 'non-coding' complementary DNAs. Nature 431: 1 p following 757; discussion following 757. 15495343

[7] Struhl K (2007) Transcriptional noise and the fidelity of initiation by RNA polymerase II. Nat Struct Mol Biol 14: 103–105. 17277804 10.1038/nsmb0207-103

[8] Ebisuya M, Yamamoto T, Nakajima M, Nishida E (2008) Ripples from neighbouring transcription. Nat Cell Biol 10: 1106–1113. 19160492 10.1038/ncb1771

[9] Mercer TR, Dinger ME, Mattick JS (2009) Long non-coding RNAs: insights into functions. Nat Rev Genet 10: 155–159. 19188922 10.1038/nrg2521

[10] Rinn JL, Kertesz M, Wang JK, Squazzo SL, Xu X, et al (2007) Functional demarcation of active and silent chromatin domains in human HOX loci by noncoding RNAs. Cell 129: 1311–1323. 17604720 10.1016/j.cell.2007.05.022 PMC2084369

[11] Loewer S, Cabili MN, Guttman M, Loh YH, Thomas K, et al (2010) Large intergenic non-coding RNA-RoR modulates reprogramming of human induced pluripotent stem cells. Nat Genet 42: 1113–1117. 21057500 10.1038/ng.710 PMC3040650

[12] Perez DS, Hoage TR, Pritchett JR, Ducharme-Smith AL, Halling ML, et al (2008) Long, abundantly expressed non-coding transcripts are altered in cancer. Hum Mol Genet 17: 642–655. 18006640 10.1093/hmg/ddm336

[13] Maruyama R, Shipitsin M, Choudhury S, Wu Z, Protopopov A, et al (2012) Altered antisense-to-sense transcript ratios in breast cancer. Proc Natl Acad Sci U S A 109: 2820–2824. 21098291 10.1073/pnas.1010559107 PMC3286925

[14] Zhang J, Zhang A, Wang Y, Liu N, You Y, et al (2012) New insights into the roles of ncRNA in the STAT3 pathway. Future Oncol 8: 723–730. 22764770 10.2217/fon.12.52

[15] Gibb EA, Brown CJ, Lam WL (2011) The functional role of long non-coding RNA in human carcinomas. Mol Cancer 10: 38. 21489289 10.1186/1476-4598-10-38 PMC3098824

[16] Wapinski O, Chang HY (2011) Long noncoding RNAs and human disease. Trends Cell Biol 21: 354–361. 21550244 10.1016/j.tcb.2011.04.001

[17] Deng Q, He B, Gao T, Pan Y, Sun H, et al (2014) Up-Regulation of 91H Promotes Tumor Metastasis and Predicts Poor Prognosis for Patients with Colorectal Cancer. PLoS ONE 9: e103022. 25058480 10.1371/journal.pone.0103022 PMC4109963

[18] Gupta RA, Shah N, Wang KC, Kim J, Horlings HM, et al (2010) Long non-coding RNA HOTAIR reprograms chromatin state to promote cancer metastasis. Nature 464: 1071–1076. 20393566 10.1038/nature08975 PMC3049919

[19] Yang Z, Zhou L, Wu LM, Lai MC, Xie HY, et al (2011) Overexpression of long non-coding RNA HOTAIR predicts tumor recurrence in hepatocellular carcinoma patients following liver transplantation. Ann Surg Oncol 18: 1243–1250. 21327457 10.1245/s10434-011-1581-y

[20] Kogo R, Shimamura T, Mimori K, Kawahara K, Imoto S, et al (2011) Long noncoding RNA HOTAIR regulates polycomb-dependent chromatin modification and is associated with poor prognosis in colorectal cancers. Cancer Res 71: 6320–6326. 21862635 10.1158/0008-5472.CAN-11-1021

[21] Chen FJ, Sun M, Li SQ, Wu QQ, Ji L, et al (2013) Upregulation of the long non-coding RNA HOTAIR promotes esophageal squamous cell carcinoma metastasis and poor prognosis. Mol Carcinog 52: 908–915. 24151120 10.1002/mc.21944

[22] Yan TH, Lu SW, Huang YQ, Que GB, Chen JH, et al. (2014) Upregulation of the long noncoding RNA HOTAIR predicts recurrence in stage Ta/T1 bladder cancer. Tumour Biol. 10.1007/s13277-014-2344-8 25030736

[23] Huang L, Liao LM, Liu AW, Wu JB, Cheng XL, et al (2014) Overexpression of long noncoding RNA HOTAIR predicts a poor prognosis in patients with cervical cancer. Arch Gynecol Obstet 10.1007/s00404-014-3236-2 24748337

[24] Svoboda M, Slyskova J, Schneiderova M, Makovicky P, Bielik L, et al (2014) HOTAIR long non-coding RNA is a negative prognostic factor not only in primary tumors, but also in the blood of colorectal cancer patients. Carcinogenesis 35: 1510–1515. 24583926 10.1093/carcin/bgu055

[25] Lu L, Zhu G, Zhang C, Deng Q, Katsaros D, et al (2012) Association of large noncoding RNA HOTAIR expression and its downstream intergenic CpG island methylation with survival in breast cancer. Breast Cancer Res Treat 136: 875–883. 23124417 10.1007/s10549-012-2314-z

[26] Liberati A, Altman DG, Tetzlaff J, Mulrow C, Gotzsche PC, et al (2009) The PRISMA statement for reporting systematic reviews and meta-analyses of studies that evaluate health care interventions: explanation and elaboration. PLoS Med 6: e1000100. 19621070 10.1371/journal.pmed.1000100 PMC2707010

[27] Izzo JG, Malhotra U, Wu TT, Ensor J, Luthra R, et al (2006) Association of activated transcription factor nuclear factor kappab with chemoradiation resistance and poor outcome in esophageal carcinoma. J Clin Oncol 24: 748–754. 16401681 10.1200/JCO.2005.03.8810

[28] Wu ZH, Wang XL, Tang HM, Jiang T, Chen J, et al (2014) Long non-coding RNA HOTAIR is a powerful predictor of metastasis and poor prognosis and is associated with epithelial-mesenchymal transition in colon cancer. Oncol Rep 32: 395–402. 24840737 10.3892/or.2014.3186

[29] Yan T-H, Lu S-W, Huang Y-Q, Que G-B, Chen J-H, et al (2014) Upregulation of the long noncoding RNA HOTAIR predicts recurrence in stage Ta/T1 bladder cancer. Tumor Biology 10.1007/s13277-014-2344-8 25030736

[30] Wang W, Chen Y, Deng J, Zhou J, Zhou Y, et al (2014) The prognostic value of CD133 expression in non-small cell lung cancer: a meta-analysis. Tumour Biol 10.1007/s13277-014-2270-9 24973892

[31] Parmar MK, Torri V, Stewart L (1998) Extracting summary statistics to perform meta-analyses of the published literature for survival endpoints. Stat Med 17: 2815–2834. 9921604 10.1002/(sici)1097-0258(19981230)17:24<2815::aid-sim110>3.0.co;2-8

[32] DerSimonian R, Laird N (1986) Meta-analysis in clinical trials. Control Clin Trials 7: 177–188. 3802833 10.1016/0197-2456(86)90046-2

[33] Mantel N, Haenszel W (1959) Statistical aspects of the analysis of data from retrospective studies of disease. J Natl Cancer Inst 22: 719–748. 13655060

[34] Sun M, Jin FY, Xia R, Kong R, Li JH, et al (2014) Decreased expression of long noncoding RNA GAS5 indicates a poor prognosis and promotes cell proliferation in gastric cancer. BMC Cancer 14: 319. 24884417 10.1186/1471-2407-14-319 PMC4022532

[35] Necsulea A, Soumillon M, Warnefors M, Liechti A, Daish T, et al (2014) The evolution of lncRNA repertoires and expression patterns in tetrapods. Nature 505: 635–640. 24463510 10.1038/nature12943

[36] Woo CJ, Kingston RE (2007) HOTAIR lifts noncoding RNAs to new levels. Cell 129: 1257–1259. 17604716 10.1016/j.cell.2007.06.014

[37] Richly H, Aloia L, Di Croce L (2011) Roles of the Polycomb group proteins in stem cells and cancer. Cell Death Dis 2: e204. 21881606 10.1038/cddis.2011.84 PMC3186902

[38] Kim K, Jutooru I, Chadalapaka G, Johnson G, Frank J, et al (2013) HOTAIR is a negative prognostic factor and exhibits pro-oncogenic activity in pancreatic cancer. Oncogene 32: 1616–1625. 22614017 10.1038/onc.2012.193 PMC3484248

[39] Milhem MM, Knutson T, Yang S, Zhu D, Wang X, et al (2011) Correlation of MTDH/AEG-1 and HOTAIR Expression with Metastasis and Response to Treatment in Sarcoma Patients. J Cancer Sci Ther S5. PMC3612017 23543869

[40] Lu L, Zhu G, Zhang C, Deng Q, Katsaros D, et al (2012) Association of large noncoding RNA HOTAIR expression and its downstream intergenic CpG island methylation with survival in breast cancer. Breast Cancer Research and Treatment 136: 875–883. 23124417 10.1007/s10549-012-2314-z

[41] Li D, Feng J, Wu T, Wang Y, Sun Y, et al (2013) Long Intergenic Noncoding RNA HOTAIR Is Overexpressed and Regulates PTEN Methylation in Laryngeal Squamous Cell Carcinoma. The American Journal of Pathology 182: 64–70. 23141928 10.1016/j.ajpath.2012.08.042

[42] Nie Y, Liu X, Qu S, Song E, Zou H, et al (2013) Long non-coding RNAHOTAIRis an independent prognostic marker for nasopharyngeal carcinoma progression and survival. Cancer Science 104: 458–464. 23281836 10.1111/cas.12092 PMC7657223

[43] Nakagawa T, Endo H, Yokoyama M, Abe J, Tamai K, et al (2013) Large noncoding RNA HOTAIR enhances aggressive biological behavior and is associated with short disease-free survival in human non-small cell lung cancer. Biochemical and Biophysical Research Communications 436: 319–324. 23743197 10.1016/j.bbrc.2013.05.101

[44] Lv XB, Lian GY, Wang HR, Song E, Yao H, et al (2013) Long noncoding RNA HOTAIR is a prognostic marker for esophageal squamous cell carcinoma progression and survival. PLoS ONE 8: e63516. 23717443 10.1371/journal.pone.0063516 PMC3662674

[45] Li X, Wu Z, Mei Q, Guo M, Fu X, et al (2013) Long non-coding RNA HOTAIR, a driver of malignancy, predicts negative prognosis and exhibits oncogenic activity in oesophageal squamous cell carcinoma. British Journal of Cancer 109: 2266–2278. 24022190 10.1038/bjc.2013.548 PMC3798955

[46] Ge X-S, Ma H-J, Zheng X-H, Ruan H-L, Liao X-Y, et al (2013) HOTAIR, a prognostic factor in esophageal squamous cell carcinoma, inhibits WIF-1 expression and activates Wnt pathway. Cancer Science 104: 1675–1682. 24118380 10.1111/cas.12296 PMC7653522

[47] Liu XH, Liu ZL, Sun M, Liu J, Wang ZX, et al (2013) The long non-coding RNA HOTAIR indicates a poor prognosis and promotes metastasis in non-small cell lung cancer. BMC Cancer 13: 464. 24103700 10.1186/1471-2407-13-464 PMC3851855

[48] Chen F-J, Sun M, Li S-Q, Wu Q-Q, Ji L, et al (2013) Upregulation of the long non-coding rna hotair promotes esophageal squamous cell carcinoma metastasis and poor prognosis. Molecular Carcinogenesis 52: 908–915. 24151120 10.1002/mc.21944

[49] Zhang JX, Han L, Bao ZS, Wang YY, Chen LY, et al (2013) HOTAIR, a cell cycle-associated long noncoding RNA and a strong predictor of survival, is preferentially expressed in classical and mesenchymal glioma. Neuro-Oncology 15: 1595–1603. 24203894 10.1093/neuonc/not131 PMC3829598

[50] Sørensen KP, Thomassen M, Tan Q, Bak M, Cold S, et al (2013) Long non-coding RNA HOTAIR is an independent prognostic marker of metastasis in estrogen receptor-positive primary breast cancer. Breast Cancer Research and Treatment 142: 529–536. 24258260 10.1007/s10549-013-2776-7

[51] Svoboda M, Slyskova J, Schneiderova M, Makovicky P, Bielik L, et al (2014) HOTAIR long non-coding RNA is a negative prognostic factor not only in primary tumors, but also in the blood of colorectal cancer patients. Carcinogenesis 35: 1510–1515. 24583926 10.1093/carcin/bgu055

[52] Qiu J-j, Lin Y-y, Ye L-c, Ding J-x, Feng W-w, et al (2014) Overexpression of long non-coding RNA HOTAIR predicts poor patient prognosis and promotes tumor metastasis in epithelial ovarian cancer. Gynecologic Oncology 134: 121–128. 24662839 10.1016/j.ygyno.2014.03.556

[53] Huang L, Liao L-M, Liu A-W, Wu J-B, Cheng X-L, et al (2014) Overexpression of long noncoding RNA HOTAIR predicts a poor prognosis in patients with cervical cancer. Archives of Gynecology and Obstetrics 10.1007/s00404-014-3236-2 24748337

[54] Liu X-h, Sun M, Nie F-q, Ge Y-b, Zhang E-b, et al (2014) Lnc RNA HOTAIR functions as a competing endogenous RNA to regulate HER2 expression by sponging miR-331-3p in gastric cancer. Molecular Cancer 13: 92. 24775712 10.1186/1476-4598-13-92 PMC4021402

[55] Wu Z-H, Wang X-L, Tang H-M, Jiang T, Chen J, et al (2014) Long non-coding RNA HOTAIR is a powerful predictor of metastasis and poor prognosis and is associated with epithelial-mesenchymal transition in colon cancer. Oncology Reports 10.3892/or.2014.3186 24840737

