# Supplementary figures and images for: Prognostic Value of Long Non-Coding RNA HOTAIR in Various Cancers
Source: PLoS One. 2014 Oct 10;9(10):e110059. doi: 10.1371/journal.pone.0110059 (PMC4193855; doi:10.1371/journal.pone.0110059)

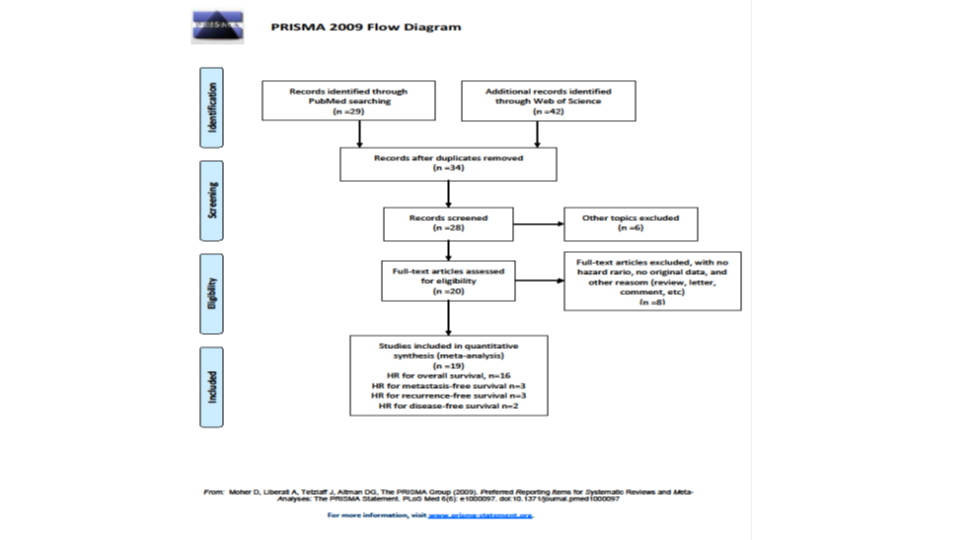

Supplement: Figure S1 — Flow chart. (TIF) [file pone.0110059.s001.tif]

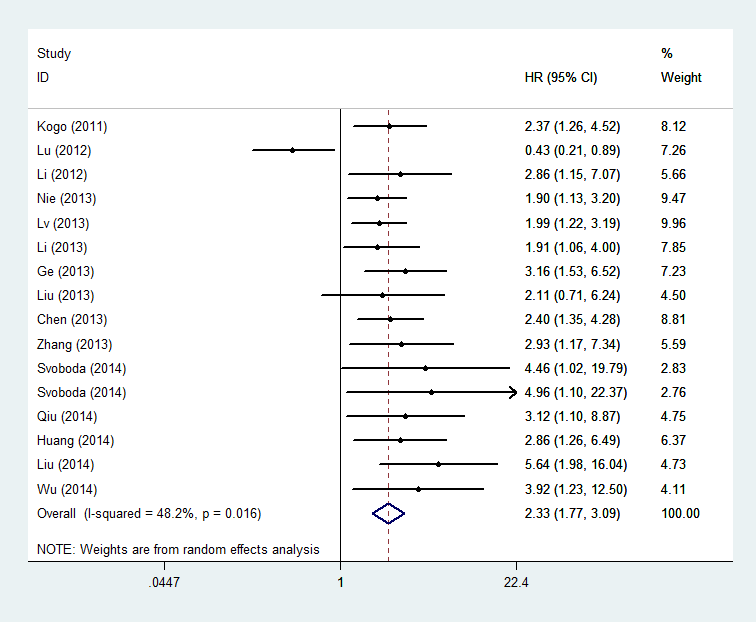

Supplement: Figure S2 — Forest plots. (TIF) [file pone.0110059.s002.tif]

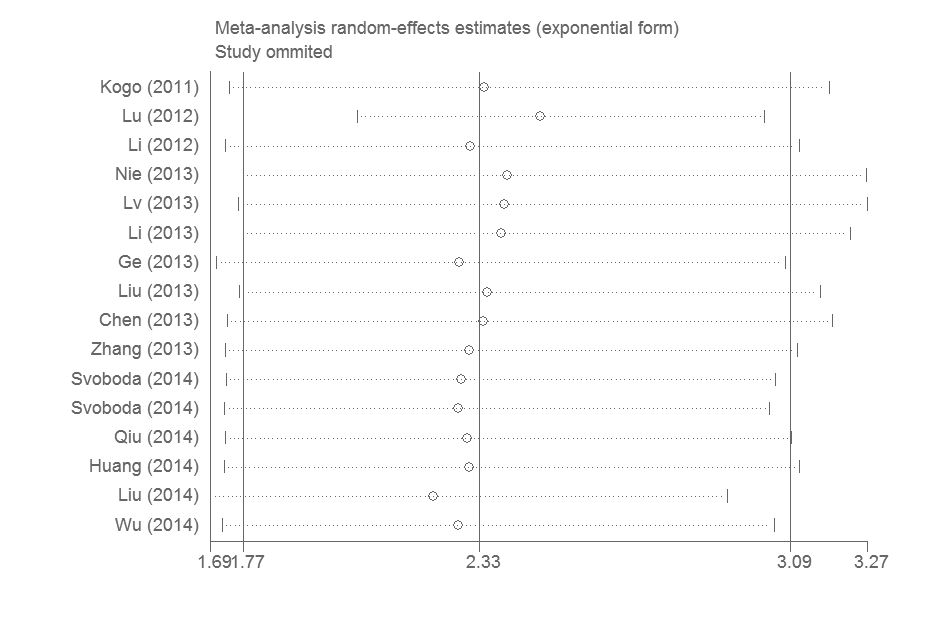

Supplement: Figure S3 — Sensitivity analysis. (TIF) [file pone.0110059.s003.tif]

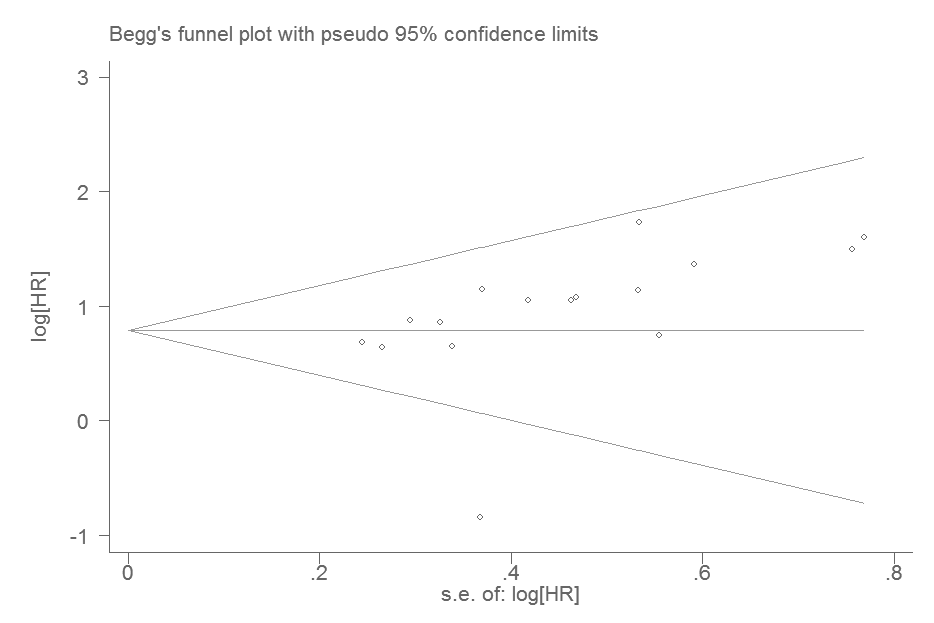

Supplement: Figure S4 — Funnel plot. (TIF) [file pone.0110059.s004.tif]
